# The New Great Northern Central Hospital

**Published:** 1888-04-21

**Authors:** 


### THE NEW GREAT NORTHERN CENTRAL HOSPITAL.

The buildings, which were opened for the reception of patients on Monday last, are a portion only of the complete scheme for the new hospital. They comprise a pavilion of wards, three floors in height, a portion of the administrative offices, the out-patient department, and the mortuary building. The ward block has on each floor a general ward for twenty beds with a duty room, small one-bed ward, linen and clothes cupboards, food cupboard, and bath-rooms, w.c.'s, lavatories, &c. The floor space in the large wards is 123·3 per bed, and the cubic space 1,613 feet. In each ward are two pairs of Boyd's hygiastic grates placed back to back in the centre of the ward; the flues from these stoves first descend, and then run under the floor to the outer wall, where they ascend. Outside, the flues are all continued downwards to the basement level, and sweep doors are provided so that they can be swept without entering the wards. The floors are constructed of iron joists and concrete, and laid with oak blocks wax polished. The roof of the ward pavilion is flat and paved with asphalte, and is intended to be used as an airing-ground for patients. The whole of the ward building is raised some eight or nine feet above the general ground level, and the space under the wards is open at the sides and has a paved floor. The immediate object of this arrangement was to raise the lowest floor of wards sufficiently above the influence of surrounding buildings to obtain ample light and air. It has the further advantage of securing a more ample circulation of air around and under the wards than would otherwise be the case, and of lifting them more completely above the influences of ground air. The administrative block consists of two parts, which are separated from one another by the corridor, which in the future will join the two ward pavilions. The front part contains, on the basement, the secretary's office and the general stores; on the ground floor, the surgery, with two retiring-rooms attached, porter's office, entrance, and medical officer's sitting-room. The first and second floors contain bed-rooms for house surgeon and physician, matron and nurses. The mezzanine formed between the second and third floors contains servants' bed-rooms. The kitchen, offices, and servants' mess-room are on the third floor. The kitchen is fitted with J. Slater and Co.'s gas cooking and steam apparatus. Two lifts worked by hydraulic power are in this portion of the building for carrying coals and food, etc. The arrangement of all the rooms in this building, except the stores, surgery, and kitchen offices, is temporary only, and will all be altered when the front administrative block is erected. The back part of this block contains the staircase and the passenger lift. In the basement are wine cellars, linen store, and porters' sitting-room. On the ground floor are two rooms destined respectively for nurses' sitting-room and dining-room, a pantry being attached to the latter. The former is to be used as a temporary board-room. On the second floor is the operating theatre, with ante-room, surgeon's lavatory, and consulting-room attached. The out-patient department is placed in a separate building, one storey in height. There are two entrances, one for men, the other for women. Immediately inside the entrance door is a small waiting-room for new patients; the seats are so arranged that each patient must follow *en gène*, and present himself or herself at the window of the office which divides the two waiting-rooms. From the smaller waiting-rooms patients enter the large waiting-hall, where again the seats are so placed that patients will be readily classified, and must take their turn. Four consulting-rooms, each with its own private room attached, are arranged at the opposite end of the hall. On the table of each consulting-room is an electric bell-push, which rings a bell in the waiting-hall, and also indicates the room in which the bell is rung by a swing disc placed in an indicator and distinctly visible to every patient in the hall. In addition to the consulting-room there is a dark room for ophthalmoscope work. From the consulting-rooms patients pass out into a corridor, and thence into a small waiting-room to the dispensary. Here, again, the same arrangement of seats compels patients to keep to their turns. The dispensary is a sky-lighted room, fitted up by Messrs. Hawke and Son. Attached to it is a private room, w.c., and lavatory for the dispenser; and in the base ment is a drug store. A telephone connects the dispensary with the surgery. There are also two additional rooms intended for the use of the electrician. The mortuary building contains a dead-house, a mortuary chamber, in which the body of a patient would be placed when seen by friends and relatives; post-mortem room, pathologist's room, and small museum. The building work generally has been carried out by Messrs. Brass and Son, the plumbers' work by Messrs. Dent and Etellger, boilers and hot-water work by Messrs. J. Slater and Co., gas-fittings by Messrs. Richardson and Ellson, and lifts by Messrs. T. Middleton and Co. The whole of the furniture has been specially made under the architects' direction by Messrs. Jenks and Wood. The architects are Messrs. Keith Young and Hall, who are entitled by the work they have done during the last few years to be placed in the first rank of hospital architects. The buildings still to be erected to complete the hospital consist of a block of circular wards, three in number, of exactly the same area as the already completed wards; and the front block, which will give accommodation to twenty-four paying patients, in addition to the permanent accommodation for the resident officers.

